# Highly precise breakpoint detection of chromosome balanced translocation in chronic myelogenous leukaemia: Case series

**DOI:** 10.1111/jcmm.17500

**Published:** 2022-07-28

**Authors:** Chuanchun Yang, Xiaoli Cui, Lei Xu, Qian Zhang, Shanmei Tang, Mengmeng Zhang, Ni Xie

**Affiliations:** ^1^ Guangdong Medical University Zhanjiang China; ^2^ CheerLand Biological Technology Co., Ltd Shenzhen China; ^3^ Department of Hematology Peking University Shenzhen Hospital Shenzhen China; ^4^ Shenzhen Second People's Hospital Shenzhen China

**Keywords:** balanced translocation, low‐coverage whole genome sequencing, Philadelphia chromosome, precise breakpoints

## Abstract

Chronic myelogenous leukaemia (CML) has a special phenomenon of chromosome translocation, which is called Philadelphia chromosome translocation. However, the detailed connection of this structure is troublesome and expensive to be identified. Low‐coverage whole genome sequencing (LCWGS) could not only detect the previously unknown chromosomal translocation, but also provide the breakpoint candidate small region (with an accuracy of ±200 bases). Importantly, the sequencing cost of LCWGS is about US$300. Then, with the Sanger DNA sequencing, the precise breakpoint can be determined at a single base level. In our project, with LCWGS, BCR and ABL1 are successfully identified to be disrupted in three CML patients (at chr22:23,632,356 and chr9:133,590,450; chr22:23,633,748 and chr9:133,635,781; chr22: 23,631,831 and chr9:133,598,513, respectively). Due to the reconnection after chromosome breakage, classical fusion gene (BCR::ABL1) was found in bone marrow and peripheral blood. The precise breakpoints were helpful to investigate the pathogenic mechanism of CML and could better guide the classification of CML subtypes. This LCWGS method is universal and can be used to detect all diseases related to chromosome variation, such as solid tumours, liquid tumours and birth defects.

## 1 INTRODUCTION

Next generation sequencing (NGS) has developed rapidly and was widely used in the field of molecular genetics.^1^ LCWGS could conduct a comprehensive detection of abnormal chromosome structure, including deletion, duplication, translocation, inversion and more complex types after their combination.^2^

Leukaemia had a high mortality rate and Chronic myeloid leukaemia (CML) accounts for 15%–20% of all adult's leukaemias.^3^, ^4^ About 90% of CML were accompanied by t(9;22)(q34;q11), which formed its iconic Philadelphia chromosome,^5^ and since DNA structure was damaged, it was often accompanied by abnormal structure of other chromosomes. In CML patients, the subtypes of BCR::ABL1 gene fusion were different. Among them, (1) >90% of patients had breakpoints in the BCR gene in exon 12–16 main break region, the resulting fusion gene protein was p210. (2) The rare BCR breakpoint occurred in the region of exon 17–20, resulting in a p230 fusion protein. (3) In rare patients, the BCR breakpoint occurred in the rare zone of exons 1–2, resulting in the fusion protein p190.^6^ The p190, p210 and p230 had persistently enhanced tyrosine kinase (TK) activity which disturbed downstream signalling pathways, causing enhanced proliferation, differentiation arrest and resistance to cell death.^7^, ^8^ The most effective drug for treating Philadelphia chromosomal disease was tyrosine kinase inhibitors (TKIs) targeting the BCR::ABL1 fusion gene protein. The biggest obstacle to improving the prognosis of patients with Ph‐positive CML was drug resistance and new mutations producing from disease progression.^9^, ^10^, ^11^ Comprehensive and accurate detection of mutations in CML patients (especially BCR::ABL1 kinase domain) in treatment progress may be the key to solving these problems.^12^

The higher accuracy of the breakpoints, the more conducive to our subsequent further analysis. LCWGS has been reported as a highly accurate, cost‐effective and robust detection approach to detect all abnormal chromosome structures.^2^ In our study, we used LCWGS to characterize the breakpoints in three CML patients with Philadelphia chromosome. We successfully mapped the breakpoints, which disrupted two known genes, BCR and ABL1. For breakpoints in patient 1 (chr22:23,632,356 and chr9:133,590,450) and patient 2 (chr22:23,633,748 and chr9:133,635,781), the fusions of chr22 and chr9 located in the 13th intron of BCR and first intron of ABL1, respectively. For patient 3 (chr22: 23,631,831 and chr9:133,598,513), the fusions located on the 14th intron of BCR and the first intron of ABL1. In addition, we also found other chromosomal structural variations. Roughly, there is no difference in the main gene fusion of different CML patients. However, at a more refined level, they will have different breakpoints and show different clinical symptoms.^13^, ^14^, ^15^ These have important guiding significance for the precise medication of patients and for doctors formulating follow‐up treatment plans. More importantly, this technology could detect relevant mutations to screen out the patients with early myeloid leukaemia, so that the doctors and patients could carry out active and effective intervention and treatment.^16^, ^17^, ^18^

## 2 MATERIALS AND METHODS

### 2.1 Case selection and sample collection

We recruited three CML patients (patient 1: a 75‐year‐old man, patient 2: a 9‐year‐old girl and patient 3: a 12‐year‐old boy) and all applied the LCWGS method. All the patients had signed the informed consent and this study was approved by the Ethics Committee of the Peking University Shenzhen Hospital. The peripheral blood of patient 1 (heparin tube) was collected for karyotyping. Additionally, the bone marrow samples and peripheral blood (EDTA tube) samples were collected for genomic DNA (gDNA) extraction after anonymization, respectively.

### 2.2 Karyotyping

For the analysis of chromosome, Giemsa (GTG) band karyotyping at 550‐band level was performed in accordance with the standard laboratory protocol.

### 2.3 LCWGS

DNA Isolation Kit for Cells and Tissues and QIAamp DNA Blood Mini Kit (QIAGEN, Hilden, Germany) is used for genomic DNA extraction from peripheral blood lymphocytes and bone marrow cells. One library of bone marrow sample was constructed with insert size of ~3 kb (mate pair).

The bone marrow library was sequenced on the Illumina NovaSeq with 151‐bp paired‐end reads and a target mean coverage of >8 folds. After removing reads containing sequencing adapters and low‐quality reads, the SOAPaligner sequence alignment software (http://soap.genomics.org.cn/) was used for mapping reads to the NCBI human reference genome (version: GRCh37.1). Then, we retained the uniquely mapped reads for the subsequent analysis and the specific analysis method has been previously described in detail. Using this specific analysis method, we could take advantage of uniquely paired reads to find all chromosome copy number variations (CNV) and structure variations (SV), and the corresponding breakpoints on the whole genome, and the accuracy of the breakpoints could be accurate to a small region of ±200 bases.

At last, accurate verification of breakpoints was carried out by Sanger sequence. We designed primers with NCBI Primer‐Blast (http://www.ncbi.nlm.nih.gov/tools/primer‐blast/) for the 500 bp upstream and 500 bp downstream of the breakpoint region respectively. By comparing the amplified products of Sanger sequence, we could determine the precise breakpoint easily. Oligonucleotide primer pairs of the translocation were designed with Gene Runner software (version 5.0.69 Beta; Hastings Software) (Table 1).

**TABLE 1:** Primer pairs for three CML patients

Cases	Fusion gene	Forword primer	Reverse primer
Patient 1	BCR::ABL1	CTAGCCTGAAGGCTGATCCC	AAGCCACTGGCACACTTCA
	ABL1::BCR	AGGGCTTTAGTTTCCTGAGGG	CAAAATCAACCATCCGGTGGAC
Patient 2	BCR::ABL1	GAGCAATACAGCGTGACACC	GCCAAAGGCTGTGAATGGTCATA
	ABL1::BCR	GCTTAGGCAATCCTCCCACTTC	CCAGGCAGCCAGAGATGACTA
Patient 3	BCR::ABL1	CTATCCTGCCCCCATCACCT	GCATTATGCTGGGGAAACAGA
	ABL1::BCR	TGATGTGTTGTGAAGTGTGTTGC	GCTTCAAAATCAACCATCCGGT

### 2.4 PCR and sanger sequencing

With designed primers, the putative fragments were amplified through PCR with general PCR conditions. The products were sequenced on an ABI‐A3130 genetic analyser.

## 3 RESULTS

**FIGURE 1:**
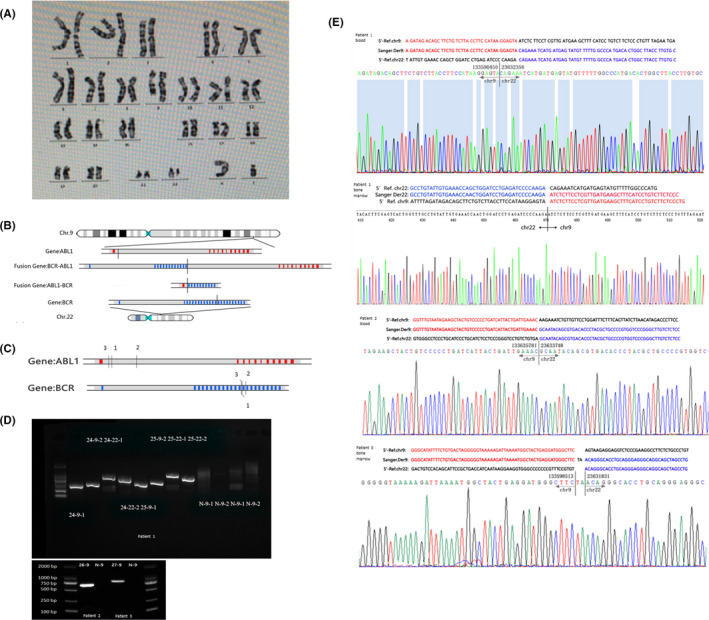
(A) Karyotype in peripheral blood for patient 1. (B) Schematic diagram of chromosome balanced translocation in bone marrow of patient 1. The detailed connection mode of the gene fusions is shown in the middle. (C) The schematic picture of breakpoints for ABL1 and BCR genes was also showed in patient 1/2/3. (D) Agarose gel map. Left: Sample 24 (bone marrow from patient 1), sample 25 (blood from patient 1). Right: sample 26 (blood from patient 2), sample 27 (bone marrow from patient 3). Negative sample (N). Two pair primers (*‐9–1 and *‐9–2, *‐22–1 and *‐22–2) were designed for the two gene fusions, BCR‐ABL1 and ABL1‐BCR. (E) ABL1::BCR's breakpoint electropherograms of Sanger sequencing: Gene fusion for patient 1 (blood sample and bone marrow sample), patient 2 (blood sample) and patient 3 (bone marrow sample)

Karyotype analysis for Patient 1’s peripheral blood indicated that he was 46,XY, t(9;22)(q34;q11.2) (Figure 1A). Due to the occurrence of balanced translocation, two fusion genes (BCR::ABL1 and ABL1::BCR) were identified. In the subsequent RT‐PCR experiment, Philadelphia chromosome (Ph) (+) was confirmed to be positive with the resulting fusion gene protein p210.

LCWGS was subsequently performed on the bone marrow sample of Patient 1 and two derivative chromosomes (der 9 and der 22) were successfully detected (Figure 1B), which identified the breakpoint on chromosome 9 in a 400 bp region (chr9:133,590,268‐133,590,668), the chromosome 22’s breakpoint in a 69 bp region (chr22: 23,632,287‐ 23,632,356) in his bone marrow sample. The same experimental analysis method was applied to the following two samples (patient 2 and 3). The schematic picture of breakpoints for ABL1 and BCR genes was also showed in patients 1/2/3 (Figure 1C). The precise position of the breakpoints was confirmed through PCR and Sanger sequencing in bone marrow samples and peripheral blood (EDTA tube) samples of the three patients (Figure 1D). As shown in Figure 1E, two accurate breakpoints of Philadelphia chromosome were the same position, chr22:23,632,356 and chr9:133,590,450 from the two different samples for patient 1. The accurate breakpoints positions were chr22:23,633,748 and chr9:133,635,781, chr22: 23,631,831 and chr9:133,598,513 for blood sample in patient 2 and bone marrow sample of patient 3, respectively.

LCWGS analysis of these cases revealed for us more results (Table 2). For patient 1, in addition to t(9;22)(q34;q11.2), we also found four CNVs: one deletion region which copy number is 1 from chr7:110933409 to chr7:111013054 (involving the IMMP2L gene), two duplication region which copy number is 3 from chr18:63,892,542 to chr18:64,158,074 (not involving the gene) and from chr18:66,308,883 to chr18:66,574,415 (involving the four genes CCDC102B, RNU6‐39P, SDHCP1, TMX3), respectively, and one duplication region which copy number is 4 from chr22:25,652,709 to chr18:25,918,145 (involving the 5 genes CRYBB2P1, IGLL3P, IGLVIVOR22‐1, LRP5L and MIR6817). There is no CNVs for patient 2, and only 1 CNV detected in patient 3 with one duplication region which copy number is 3 from chr3:186,502,068 to chr3:186,670,517 (involving the 5 genes EIF4A2, RFC4, ADIPOQ and ST6GAL1). Any gene with CNV reported from the case in those previous reported studies of PubMed or in OMIM or in DGV was considered as high confidence for a particular phenotype, and these CNVs were therefore considered to be benign.

**TABLE 2:** Chromosome CNVs for three CML patients

Cases	Chr	CNV	Mutation Type	Copy Number	Gene	OMIM ID	Pheno‐type	DGV
Patient 1	7	110,933,409–111,013,054	loss	1	IMMP2L	605,977	/	0.0006
	18	63,892,542–64,158,074	gain	3	/	/	/	0.0003
	18	66,308,883–66,574,415	gain	3	CCDC102B	/	/	0.00007
					RNU6‐39P	/	/	
					SDHCP1	/	/	
					TMX3	616,102	/	
	22	25,652,709–25,918,145	gain	4	CRYBB2P1	/	/	0.0004
					LGLL3P	/	/	
					LGLVIVOR22‐1	/	/	
					LRP5L	/	/	
					MIR6817	/	/	
Patient 2	/	/	/	/	/	/	/	/
Patient 3	3	186,502,068–186,670,517	gain	3	EIF4A2	601,102	/	0.00008
					RFC4	102,577	/	
					ADIPOQ	605,441	/	
					ST6GAL1	109,675	/	

## 4 DISCUSSION

CML originates from multipotent haematopoietic stem cells and BCR::ABL was the main driving event in CML.^19^, ^20^ A gene fusion mutation occurred between the BCR and ABL1 genes; however, the position of the connection breakpoint changed greatly.^21^ According to the different connection breakpoints of the BCR::ABL1 fusion gene, the length of the corresponding expressed protein would be different. According to this, it could be divided into P190, P210 and P230. Among them, P210 is the most common.^22^ Because the gene structure was changed in CML patients, it was often accompanied by variations of SVs and CNVs in other chromosomes.^23^ Although most of these mutations were not reported in the literature, their clinical significance was unclear. During traditional tyrosine kinase inhibitors (TKIs) treatment process, patients could develop drug resistance with poor prognosis. It might due to the occurrence of new chromosomal SVs or new BCR::ABL1 fusion subtypes.^24^, ^25^, ^26^

A lot of laboratories were currently in the process of introducing NGS into their routine diagnostic procedures, as it is shown to be a robust, reproducible and cost‐effective alternative to traditional detection methods.^27^ In this study, we successfully applied LCWGS method for the detection of chromosome translocation in CML patient series, which given the candidate region of the breakpoint, and finally by combining the results of Sanger sequencing to determine the precise breakpoint. Besides, this method could detect all chromosome SVs and CNVs in the samples. There is none CNVs in patient 2 and only 1 CNV detected in patient 3 when compared with 4 CNVs in patient 1, that might because patients 2 and 3 are much younger than patient 1. This was of great significance for the early screening of CML patients, the accompanying diagnosis during the treatment process, the discovery of new BCR::ABL1 mutation subtypes, and subsequent intervention and treatment. It had been reported in the literature that the Philadelphia chromosome of CML could be treated by gene editing, which required very high requirements for precise breakpoints of gene fusion and other possible mutations.^28^ LCWGS had high accuracy, high resolution and comprehensive detection, which happened to provide a panoramic description of chromosome genome mutations in CML patients. Our results proved that the method of precise breakpoint detection of complex chromosome rearrangement could be employed as a diagnostic tool for CML patients.

Cost is the biggest factor affecting the clinical application of a new technology. LCWGS is highly cost‐effective with a lower coverage‐depth sequencing. In this case, ~80 million paired reads (~24Gb bases) were obtained, and the cost was about US$300 per sample for using our approach. Although the sequencing cost decreases dramatically in the last few decades, the cost for WGS is still too high considering the budget.^29^ Considering screening the whole genome while remaining individual information, the per‐sample sequencing reads for LCWGS is ~80 M about 8‐fold coverage while long‐read SMRT sequencing needs ~40‐fold to find chromosome SV.^30^ Additionally, in a study of CML's cell lines, more than 60‐fold sequence coverage data were generated.^31^ Furthermore, even if we generate the same amount of data, the cost of Pacific Biosciences (PacBio) is higher than that of Illumina's NovaSeq.^32^

Next, we will continue to improve the detection accuracy and lower limit of the data abundance of the algorithm, so that it can screen out the variation types in early‐stage patients and other subtypes that are newly developed during the progression of leukaemia. Finally, it will provide guidance for gene editing therapy and the combination of targeted drugs.

## 5 CONCLUSION

LCWGS is a cost‐effective and accurate method to detect chromosome SVs and CNVs including deletion, duplication, inversion and translocation without known karyotyping result. It can play a vital role in solid tumours and liquid tumours. The premise of accurate medical treatment is accurate detection.

## AUTHOR CONTRIBUTIONS

**chuanchun yang:** Investigation (lead); methodology (lead); writing – original draft (lead). **xiaoli cui:** Software (equal). **lei xu:** Data curation (equal). **qian zhang:** Data curation (equal). **shanmei tang:** Software (equal). **mengmeng zhang:** Investigation (equal). **ni xie:** Funding acquisition (equal); project administration (equal); supervision (equal).

## FUNDING INFORMATION

This work was supported by Sanming Project of Medicine in Shenzhen(NO.SZSM201612004). This project was supported by the National Natural Science Foundation of China, China (No. 82172356, No. 81972003), the Natural Science Foundation of Guangdong, China (No. 2021A1515012144), Science, Technology and Innovation Commission of Shenzhen Municipality (No. JCYJ20180507182025817).

## CONFLICT OF INTEREST

None.

## INFORMED CONSENT

All patients provided written informed consent before participation.

## Data Availability

The original data of this project can be easily obtained from the author by e‐mail.

## References

[1] Chen W , Kalscheuer V , Tzschach A , et al. Mapping translocation breakpoints by next‐generation sequencing. Genome Res. 2008;18:1143‐1149. 18326688 10.1101/gr.076166.108 PMC2493403

[2] Dong Z , Jiang L , Yang C , et al. A robust approach for blind detection of balanced chromosomal rearrangements with whole‐genome low‐coverage sequencing. Hum Mutat. 2014;35:625‐636. 24610732 10.1002/humu.22541

[3] Chen W , Zheng R , Zeng H , Zhang S . The updated incidences and mortalities of major cancers in China, 2011. Chin J Cancer. 2015;34:502‐507. 26370301 10.1186/s40880-015-0042-6 PMC4593358

[4] Siegel RL , Miller KD , Jemal A . Cancer statistics, 2015. CA Cancer J Clin. 2015;65:5‐29. 25559415 10.3322/caac.21254

[5] Marzocchi G , Castagnetti F , Luatti S , et al. Variant Philadelphia translocations: molecular‐cytogenetic characterization and prognostic influence on frontline imatinib therapy, a GIMEMA Working Party on CML analysis. Blood. 2011;117:6793‐6800. 21447834 10.1182/blood-2011-01-328294

[6] Kang Z , Liu Y , Xu L , et al. The Philadelphia chromosome in leukemogenesis. Chin J Cancer. 2016;35:48. 27233483 10.1186/s40880-016-0108-0 PMC4896164

[7] Kurzrock R , Gutterman JU , Talpaz M . The molecular genetics of Philadelphia chromosome‐positive leukemias. N Engl J Med. 1988;319:990‐998. 3047582 10.1056/NEJM198810133191506

[8] Li, S. , R. Ilaria , R. Million , G Daley Q , Van Etten RA . The P190, P210, and P230 forms of the BCR/ABL oncogene induce a similar chronic myeloid leukemia–like syndrome in mice but have different lymphoid leukemogenic activity. J Exp Med (1999) 189:1399–412. 10224280 10.1084/jem.189.9.1399 PMC2193055

[9] Ottmann OG , Druker BJ , Sawyers CL , et al. A phase 2 study of imatinib in patients with relapsed or refractory Philadelphia chromosome‐positive acute lymphoid leukemias. Blood. 2002;100:1965‐1971. 12200353 10.1182/blood-2001-12-0181

[10] Sawyers CL , Hochhaus A , Feldman E , et al. Imatinib induces hematologic and cytogenetic responses in patients with chronic myelogenous leukemia in myeloid blast crisis: results of a phase II study. Blood. 2002;99:3530‐3539. 11986204 10.1182/blood.v99.10.3530

[11] Tojo A , Usuki K , Urabe A , et al. A Phase I/II study of nilotinib in Japanese patients with imatinib‐resistant or ‐intolerant Ph+ CML or relapsed/refractory Ph+ ALL. Int J Hematol. 2009;89:679‐688. 19449194 10.1007/s12185-009-0327-0

[12] Corbin AS , Agarwal A , Loriaux M , Cortes J , Deininger MW , Druker BJ . Human chronic myeloid leukemia stem cells are insensitive to imatinib despite inhibition of BCR‐ABL activity. J Clin Invest. 2011;121:396‐409. 21157039 10.1172/JCI35721 PMC3007128

[13] Verrma SP , Dutta TK , Vinod KV , Dubashi B , Ariga KK . Philadelphia chromosome positive pre‐T cell acute lymphoblastic leukemia: a rare case report and short review. Indian J Hematol Blood Transfus. 2014;30:177‐179. 25332571 10.1007/s12288-013-0314-8 PMC4192242

[14] Rafiei A , Mian AA , Doring C , et al. The functional interplay between the t(9;22)‐associated fusion proteins BCR/ABL and ABL/BCR in Philadelphia chromosome‐positive acute lymphatic leukemia. PLoS Genet. 2015;11:e1005144. 25919613 10.1371/journal.pgen.1005144 PMC4412790

[15] Zhang LJ , Gan YM , Yu L . Occurrence of BCR/ABL fusion gene in a patient with acute promyelocytic leukemia. Med Oncol. 2015;32:382. 25428388 10.1007/s12032-014-0382-0

[16] Choi W , Kim M , Lim J , et al. Four cases of chronic myelogenous leukemia in mixed phenotype blast phase at initial presentation mimicking mixed phenotype acute leukemia with t(9;22). Ann Lab Med. 2014;34:60‐63. 24422198 10.3343/alm.2014.34.1.60 PMC3885775

[17] Matutes E , Pickl WF , Van't Veer M , et al. Mixed‐phenotype acute leukemia: clinical and laboratory features and outcome in 100 patients defined according to the WHO 2008 classification. Blood. 2011;117:3163‐3171. 21228332 10.1182/blood-2010-10-314682

[18] Mack EKM , Marquardt A , Langer D , et al. Comprehensive genetic diagnosis of acute myeloid leukemia by next‐generation sequencing. Haematologica. 2019;104(2):277‐287. 30190345 10.3324/haematol.2018.194258 PMC6355503

[19] Peng Y , Pang J , Hu J , et al. Clinical and molecular characterization of 12 prenatal cases of Cri‐du‐chat syndrome. Mol Genet Genomic Med. 2020;8:e1312. 32500674 10.1002/mgg3.1312 PMC7434726

[20] Bacher U , Haferlach T , Alpermann T , et al. Subclones with the t(9;22)/BCR‐ABL1 rearrangement occur in AML and seem to cooperate with distinct genetic alterations. Br J Haematol. 2011;152:713‐720. 21275954 10.1111/j.1365-2141.2010.08472.x

[21] Gary Gilliland D . Molecular genetics of human leukemias: new insights into therapy. Semin Hematol. 2002;39:6‐11. 12447846 10.1053/shem.2002.36921

[22] Sabattini E , Bacci F , Sagramoso C , Pileri SA . WHO classification of tumours of haematopoietic and lymphoid tissues in 2008: an overview. Pathologica. 2010;102:83‐87. 21171509

[23] Branford S , Wang P , Yeung DT , et al. Integrative genomic analysis reveals cancer‐associated mutations at diagnosis of CML in patients with high‐risk disease. Blood. 2018;132(9):948‐961. 29967129 10.1182/blood-2018-02-832253

[24] Voncken JW , Morris C , Pattengale P , et al. Clonal development and karyotype evolution during leukemogenesis of BCR/ABL transgenic mice. Blood. 1992;79:1029‐1036. 1737087

[25] Sill H , Goldman JM , Cross NC . Homozygous deletions of the p16 tumor‐suppressor gene are associated with lymphoid transformation of chronic myeloid leukemia. Blood. 1995;85:2013‐2016. 7718873

[26] Ilaria R Jr . Bcr/Abl, leukemogenesis, and genomic instability: a complex partnership. Leuk Res. 2002;26:971‐973. 12363461 10.1016/s0145-2126(01)00180-1

[27] Score J , Calasanz MJ , Ottman O , et al. Analysis of genomic breakpoints in p190 and p210 BCR‐ABL indicate distinct mechanisms of formation. Leukemia. 2010;24:1742‐1750. 20703256 10.1038/leu.2010.174

[28] Chen SH , Hsieh YY , Tzeng HE , et al. ABL genomic editing sufficiently abolishes oncogenesis of human chronic myeloid leukemia cells in vitro and in vivo. Cancers (Basel). 2020;12:1399. 10.3390/cancers12061399 PMC7352505 32485885

[29] Wetterstrand KA . DNA sequencing costs: data from the NHGRI genome sequencing program (GSP) Available at: www.genome.gov/sequencingcostsdata. Accessed [date of access].

[30] Chaisson MJ , Huddleston J , Dennis MY , et al. Resolving the complexity of the human genome using single‐molecule sequencing. Nature. 2015;517(7536):608‐611. 25383537 10.1038/nature13907 PMC4317254

[31] Deregowska A , Pepek M , Pruszczyk K , Machnicki MM , Wnuk M , Stoklosa T . Differential regulation of telomeric complex by BCR‐ABL1 kinase in human cellular models of chronic myeloid leukemia‐from single cell analysis to next‐generation sequencing. Genes. 2020;11(10):1145. doi:10.3390/genes11101145 PMC7601685 33003326

[32] Yoshinaga Y , Daum C , He G , O'Malley R . Genome Sequencing. Methods Mol Biol. 2018;1775:37‐52. 29876807 10.1007/978-1-4939-7804-5_4

